# Supplementary material for: The genome sequence of the white-beaked dolphin, Lagenorhynchus albirostris (Gray, 1846)
Source: Wellcome Open Res. 2024 Nov 20;9:687. [Version 1] doi: 10.12688/wellcomeopenres.23369.1 (PMC11615437; doi:10.12688/wellcomeopenres.23369.1)
Supplement: Peer review history

# Reviewer report: Reviewer response for version 1

**DOI:** 10.21956/wellcomeopenres.25764.r112640

**Published:** 2024-12-03

**Authors:** Michael V Westbury

**Affiliations:** 1 Section for Evolutionary Genomics, University of Copenhagen, Copenhagen, Denmark

This report presents a new genome assembly from a female white-beaked dolphin *Lagenorhynchus albirostris*. It was assembled using both PacBio HiFi and Illumina Hi-C data. The assembly can be considered of high quality and contiguity as most (94.46%) of the

 assembly sequence was assigned to 22 chromosomal-level scaffolds with an N50 of 111Mb. Despite this, the orientations of four regions on Chr1, 6, 8, and 12 could not be determined.

 The methodologies appear sound and suitable.

Are sufficient details of methods and materials provided to allow replication by others?

Yes

Is the rationale for creating the dataset(s) clearly described?

Yes

Are the datasets clearly presented in a useable and accessible format?

Yes

Are the protocols appropriate and is the work technically sound?

Yes

Reviewer Expertise:

I am an evolutionary geneticist with experience in genome assembly and downstream comparative and population genomic analyses. I study various mammalian species, including marine mammals.

I confirm that I have read this submission and believe that I have an appropriate level of expertise to confirm that it is of an acceptable scientific standard.

# Reviewer report: Reviewer response for version 1

**DOI:** 10.21956/wellcomeopenres.25764.r112644

**Published:** 2024-11-28

**Authors:** Jayan Duminda.M Senevirathna

**Affiliations:** 1 Uva Wellassa University, Badulla, Uva Province, Sri Lanka

In keywords, scientific name must be italic

 In methodology, explain how did you collect the specimen...? Is it live specimen or stranded...?

 The annotation results using RNA data has not been include in the manuscript. Did you use RNA data in any of  the genome development tools...? Otherwise, you can remove.

Are sufficient details of methods and materials provided to allow replication by others?

Yes

Is the rationale for creating the dataset(s) clearly described?

Yes

Are the datasets clearly presented in a useable and accessible format?

Yes

Are the protocols appropriate and is the work technically sound?

Yes

Reviewer Expertise:

Genetics, Molecular biology and biotechnology for aquatic resource management

I confirm that I have read this submission and believe that I have an appropriate level of expertise to confirm that it is of an acceptable scientific standard.
